# Significant increase in graupel and lightning occurrence in a warmer climate simulated by prognostic graupel parameterization

**DOI:** 10.1038/s41598-024-54544-5

**Published:** 2024-02-16

**Authors:** Takuro Michibata

**Affiliations:** Department of Earth Science, Okayama University, Okayama, Japan

**Keywords:** Climate model, Graupel, Lightning, Global warming, Arctic climate, Climate sciences, Atmospheric science, Climate change, Cryospheric science

## Abstract

There is little consensus among global climate models (CGMs) regarding the response of lightning flash rates to past and future climate change, largely due to graupel not being included in models. Here a two-moment prognostic graupel scheme was incorporated into the MIROC6 GCM and applied in three experiments involving pre-industrial aerosol, present-day, and future warming simulations. The new microphysics scheme performed well in reproducing global distributions of graupel, convective available potential energy, and lightning flash rate against satellite retrievals and reanalysis datasets. The global mean lightning rate increased by 7.1% from the pre-industrial period to the present day, which was attributed to increased graupel occurrence. The impact of future warming on lightning activity was more evident, with the rate increasing by 18.4$\%\,\textrm{K}^{-1}$ through synergistic contributions of destabilization and increased graupel. In the Arctic, the lightning rate depends strongly on the seasonality of graupel, emphasizing the need to incorporate graupel into GCMs for more accurate climate prediction.

## Introduction

Lightning plays a significant role in atmospheric chemistry and environmental processes by providing nitrogen oxides and initiating wildfires^1,2^. Despite the importance of climate prediction, the link between anthropogenic climate change and lightning variability remains controversial. Previous observation-based studies have shown that lightning activity has increased over the last decade^3,4^, particularly in the Arctic region^5^, although some studies have reported insignificant trends^6^. As lightning may trigger wildfires in high-latitude regions^7–9^, its increasing frequency may exacerbate future warming through the release of $\textrm{CO}_{2}$ and permafrost methane^10,11^.

Increased lightning in response to global warming is also indicated by modeling studies^12^, although some simulations indicate decreasing trends with increasing warming^13^. Lightning activity depends on various factors including atmospheric instability, aerosols, cloud depth, and the mass of ice hydrometeors^14,15^, making accurate simulation difficult^16–19^.

A limitation of global climate models (GCMs) is that they do not consider high-density ice hydrometeors (graupel and hail) in cloud microphysical parameterization. Many GCMs treat precipitation (rain, snow, and graupel/hail) diagnostically, with precipitating hydrometeors being removed from the atmosphere in a single model time-step^20^. Although a few GCMs incorporate prognostic precipitation^21,22^, little consideration has been given to the link between graupel and lightning^23^. An improved understanding of the effects of climate change on lightning activity, at a fundamental process level in GCMs, requires more sophisticated linking of cloud and precipitation parameterization to lightning.

To this end, prognostic precipitation in the Model for Interdisciplinary Research on Climate v.6 (MIROC6), was expanded here to allow for graupel hydrometeor triggering of lightning. The various lightning parameterizations applied have led to variability in model lightning evaluation^16,17^, so a function of convective available potential energy (CAPE) and column ice hydrometeors was employed, which has been shown to perform well against satellite observations^23^. The present study considered how changes in graupel and meteorology affect lightning activity through a series of sensitivity experiments involving perturbations of temperature and aerosol conditions, examining regional and seasonal characteristics with a particular focus on the Arctic, where lightning effects may accelerate global warming.

## Results

### Global climatology for graupel and lightning

**Figure 1:**
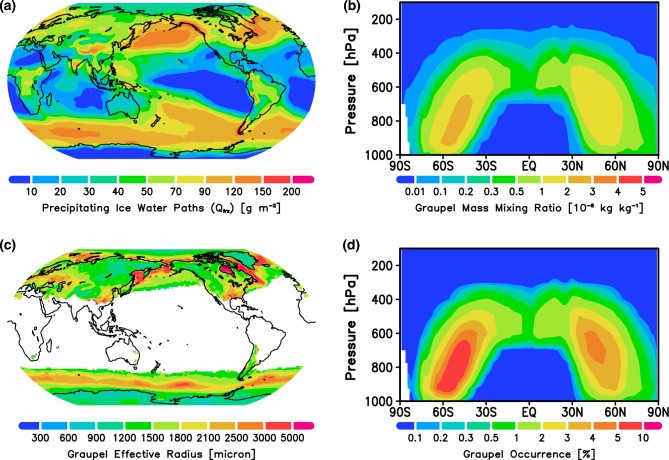
Spatial distributions of (**a**) precipitating ice water paths ($\textrm{Q}_{\textrm{frz}}$; $\textrm{g}\,\textrm{m}^{-2}$); (**b**) zonal mean graupel mass-mixing ratio ($10^{-6}\,\textrm{kg}\,\textrm{kg}^{-1}$); (**c**) graupel effective radius ($\upmu \textrm{m}$) at the surface; and (**d**) zonal mean graupel occurrence (%) simulated in the present-day experiment.

The graupel distribution introduced in the model and total ice hydrometeors should be verified before consideration of variations in lightning flash rate. Total precipitating ice hydrometeor ($\textrm{Q}_{\textrm{frz}}$) is widely distributed over the tropical eastern Indian Ocean, the Maritime Continent, and the western Pacific where deep convection occurs, and over mid-latitude oceanic regions such as the North Pacific, east coast of North America, and Southern Ocean where super-cooled liquid clouds are frequently observed (Fig. 1a,b). The global annual mean graupel water path (GWP) is 3.8 $\textrm{g}\,\textrm{m}^{-2}$, similar to that applied in other global embedded-cloud-resolving models such as Multiscale Modeling Framework models^24,25^.

The graupel effective radius at the surface is mapped in Fig. 1c. It is regime-dependent and varies widely. In general, graupel size is not simply correlated with the $\textrm{Q}_{\textrm{frz}}$, as its growth is controlled in a complex manner by various microphysical processes (e.g., increasing through riming and collection among other hydrometeors, and decreasing through melting and/or production of smaller ice nuclei), depending on the cloud and environmental regimes^26,27^. Simulated graupel size and fall velocity (Supplementary Fig. 1) vary within a reasonable observed range^28^. The modeled graupel occurrence frequency (Fig. 1d), defined by the fractional coverage of precipitation, is reasonable when compared with the other GCM that includes prognostic graupel^29^.

**Figure 2:**
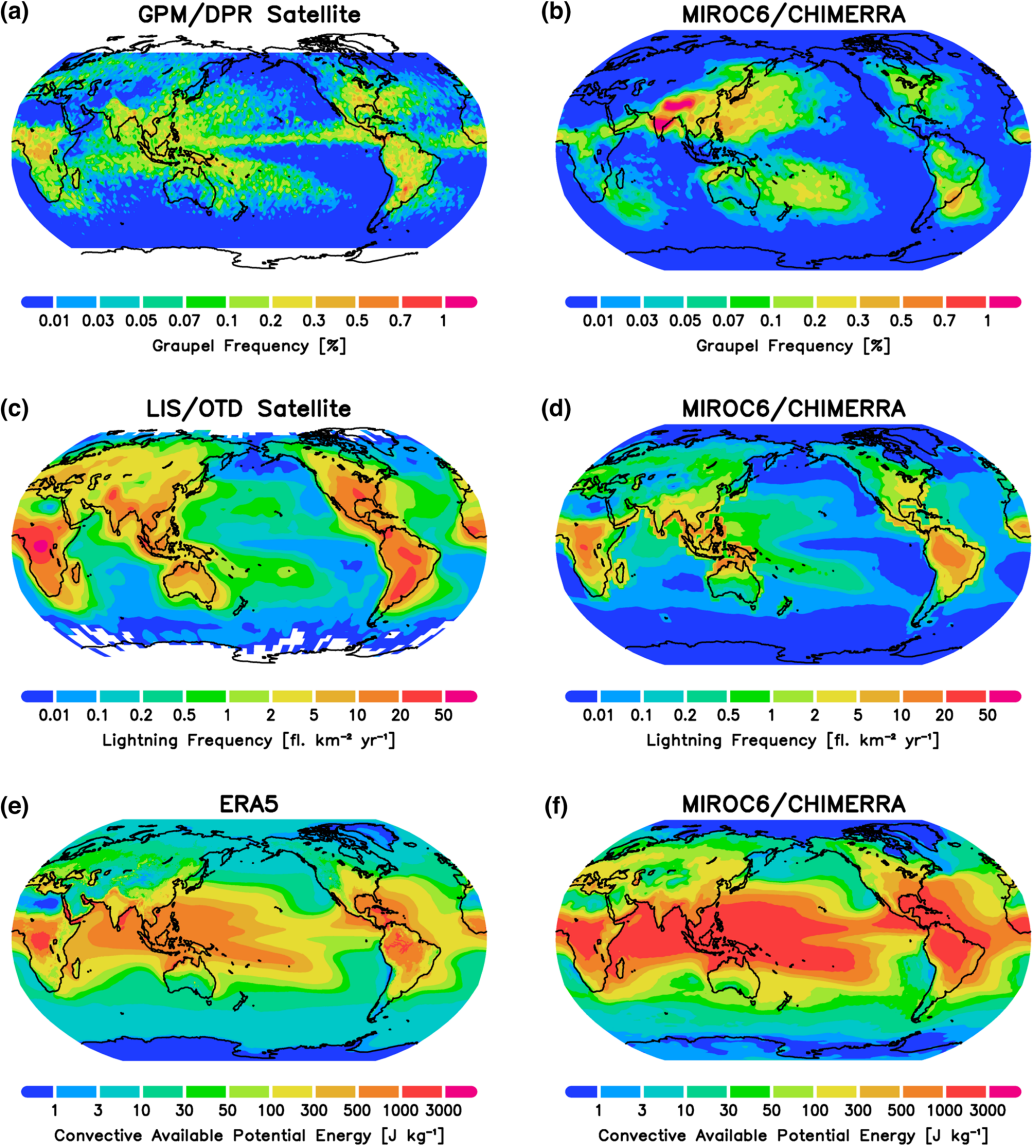
Spatial distributions of (top) the occurrence frequency of graupel (%) from (**a**) Global Precipitation Measurement Dual-frequency Precipitation Radar (GPM/DPR) retrieval for January 2021–December 2022, and (**b**) the MIROC6-CHIMERRA PD simulation; (middle) the lightning flash rate ($\textrm{fl}.\,\textrm{km}^{-2}\,\textrm{yr}^{-1}$) from (**c**) LIS/OTD satellite observations for July 1995–February 2014, and (**d**) MIROC6-CHIMERRA PD simulation; and (bottom) the convective available potential energy (CAPE; $\textrm{J}\,\textrm{kg}^{-1}$) from (**e**) ERA5 climatology for 1980–2021^30^ and (**f**) MIROC6-CHIMERRA PD simulation.

Although there are no global observational products for graupel distribution, the Global Precipitation Measurement (GPM) Dual-frequency Precipitation Radar (DPR) provides the occurrence frequency of heavy ice precipitation (e.g., graupel and hail^31^) at latitude of 65^∘^ S to 65^∘^ N^32,33^. Figure 2a shows the GPM-observed annual occurrence frequency of graupel. Since the distribution is dependent on the definition of high-density ice hydrometeors in satellite retrieval algorithms, biases are introduced when evaluating the model performance against observations (Fig. 2b). For example, there are significant errors over Europe, the Indian subcontinent, and the Qinghai–Tibet Plateau owing to the underestimated cloud coverage over land^34^. However, the model performs well in representing the overall graupel distribution.

The observed and simulated global lightning distributions are also shown in Fig. 2, in which the widely reported land–ocean contrast (Fig. 2c,d) is evident, due to lightning being triggered not only by convection associated with atmospheric instability but also to frozen hydrometeors within clouds affecting charge separation^35^. Thus, the spatial distribution of lightning is not determined by CAPE alone (Fig. 2e,f).

The present study used the lightning scheme parameterized by both CAPE and precipitating ice hydrometeors coupled to prognostic graupel [Eq. (3)], and the simulated spatial pattern of flash rate is similar to that of LIS/OTD satellite observations (Fig. 2c,d). The model captured higher lightning flash rates well, particularly over Central Africa, the Amazon, the eastern coast of North America, India, and Southeast Asia. The flash rate is somewhat underestimated over the tropical convective zones, partly because of the coarse vertical resolution and because GCMs cannot explicitly resolve convective clouds. These issues may be addressed in a future study.

**Table 1:** Global and regional mean climatology of relative changes in lightning flash rate and associated parameters from pre-industrial aerosol levels (PI, year 1850) to present-day (PD, year 2000), and from PD through future warming (SST + 4K).

	PD change relative to PI mean (Global) (%)	Future change relative to PD mean (Global) (% K^-1^)	Future change relative to PD mean (Arctic) (% K^-1^)	Future change relative to PD mean (Antarctic) (% K^-1^)
Lightning Rate	+ 7.1*	+ 18.4**	+ 12.2**	+ 26.4**
CLWP^a^	+ 1.4	+ 5.1**	+ 19.2**	+ 28.1**
CIWP^b^	+ 3.7*	+ 8.1**	+ 12.2**	+ 12.9**
GWP^c^	+ 6.0*	+ 3.0**	+ 13.8**	+ 45.0**
$\textrm{Q}_{\textrm{frz}}$^d^	+ 3.7	+ 4.3**	+ 9.0**	+ 23.1**
Graupel occurrence	+ 10.1*	+ 7.9**	+ 14.9**	+ 106.4**
Graupel radius	+ 1.9	+ 3.4**	+ 9.7**	+ 4.7**
CAPE^e^	+ 1.4	+ 9.7**	+ 7.8**	+ 5.3**

A single asterisk (*) indicates statistical significance at the 95–99% confidence level; a double asterisk (**) indicates statistical significance at the > 99% confidence level.

^a^CLWP, Cloud Liquid Water Path.

^b^CIWP, Cloud Ice Water Path.

^c^GWP, Graupel Water Path.

^d^ $\textrm{Q}_{\textrm{frz}}$: Total precipitating ice hydrometer [see Eq. (4)].

^e^CAPE, Convective Available Potential Energy.

The global mean lightning rate increased by 7.1% from the PI to PD simulations (Table 1), due mainly to increased ice hydrometeors over the tropics (Supplementary Fig. 2), implying a signal of ‘cloud invigoration hypothesis’^36^ linking changes in lightning, graupel, and aerosols^14,37–39^. The cloud liquid water path (CLWP), cloud ice water path (CIWP), and GWP increase in response to the change in aerosol from PI to PD, but the graupel is the most sensitive to this change (Table 1). This is likely because the increased cloud liquid and ice water contribute directly to the source of graupel. Given that CAPE is almost constant (Supplementary Table 1), the increased lightning rate is fundamentally attributed to the increased occurrence of graupel. The effective radiative forcing due to aerosol–cloud interactions ($\textrm{ERF}_{\textrm{aci}}$; see “Methods” section) is − 0.62 $\textrm{W}\,\textrm{m}^{-2}$ in the new MIROC6-CHIMERRA (Cloud-Hydrometeors Interactive Module with Explicit Rain and Radiation; see “Methods” section) model with prognostic graupel, $\sim$ 22% lower than that of the previous model without graupel (− 0.79 $\textrm{W}\,\textrm{m}^{-2}$). This may be explained by the ‘snow-induced ACI buffering hypothesis’^34^, which emphasizes the important role of the riming of underlying super-cooled liquid clouds by falling snowflakes and graupel at mid latitudes, effectively removing cloud water^40^ even though anthropogenic aerosols are increased from PI levels.

### Future changes in graupel and lightning

**Figure 3:**
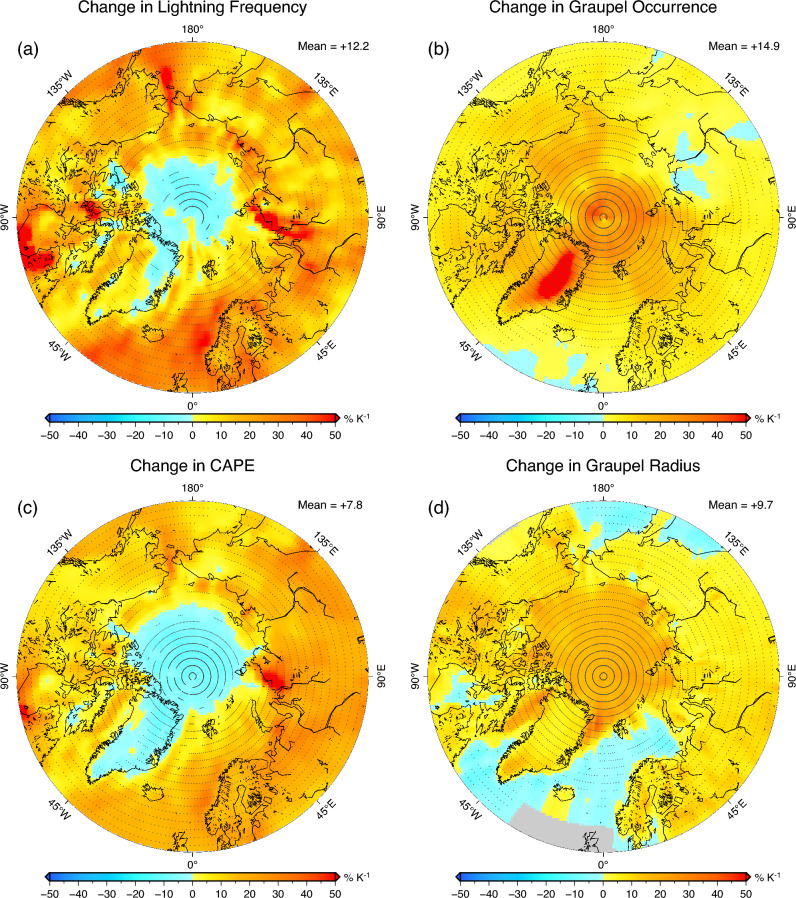
Spatial distributions (above 55^∘^ N) of future changes relative to PD ($\%\,\textrm{K}^{-1}$) in (**a**) lightning flash rate, (**b**) graupel occurrence, (**c**) CAPE, and (**d**) graupel effective radius. Dotted regions indicate a statistically significant difference at the 95% confidence level.

The response of lightning flash rate to a unit increase in global surface air temperature over higher northern latitudes (above 55^∘^ N) is shown in Fig. 3a. An increased flash rate is predicted for most regions, with a mean increase over the Arctic (66^∘^ N–90^∘^ N) of 12.2$\%\,\textrm{K}^{-1}$. The spatial pattern is inherently linked to that of graupel (Fig. 3b) and CAPE (Fig. 3c) responses [Eq. (3)]. More specifically, the distribution of the lightning change is generally similar to the change in CAPE, with the coincident increase in graupel canceling out the reduced lightning frequency due to reduced CAPE (i.e., stabilization) around the North Pole.

Although graupel and CAPE are the main controls on the lightning distribution in the model, the responses of meteorology and large-scale circulation are also important factors in lightning prediction because our simulations suggest that increased graupel is a result of incremental changes in CLWP and CIWP with increasing temperature (Table 1). Graupel size may be related to the cloud and environmental regimes (Fig. 1c), and future responses of graupel size to warming will also be independent of graupel occurrence and CAPE (Fig. 3d and Supplementary Fig. 3). The change in hydrometeor size is directly related to fall speed and thus the strength of the hydrological cycle, which may in turn be linked to charge separation within clouds^41^. Although beyond the scope of this study, an improved physical understanding of lightning occurrence and more elaborate parameterization may help to reduce model uncertainty and variability^12,16^.

The global mean sensitivities of graupel occurrence and CAPE to a unit temperature increase are comparable, + 7.9$\%\,\textrm{K}^{-1}$ and + 9.7$\%\,\textrm{K}^{-1}$, respectively (Table 1). Over polar regions, however, the contribution of change in graupel occurrence is more evident (Table 1, and Supplementary Figs. 3 and 4). As a result of the combined effects of changes in graupel and CAPE, the global mean response of lightning flash rate is + 18.4$\%\,\textrm{K}^{-1}$, higher than previous estimates of $-5$ to + 12$\%\,\textrm{K}^{-1}$^13,42^.

This increase in lightning flash rate may be attributed mainly to different model performances in representing cloud vertical structures^17,43^, different treatments of convective clouds that are not resolved in GCMs^44^, or different microphysics process representations^19^. Although the model of the present study included the treatment of graupel in large-scale condensation, a fundamental physical consideration of future lightning changes with regard to convective clouds may have been overlooked^45^. The present results indicate the need for a prognostic treatment of precipitation, including high-density ice crystallites, for both cumulus and large-scale condensation; such a GCM is not currently available.

Models and experimental configurations vary among studies in terms of, for example, future scenarios, $\textrm{CO}_{2}$ and aerosol emissions, horizontal/vertical resolution, and microphysical complexity. Although the responses of atmospheric circulation, surface forcing, and consequent feedback also differ between atmosphere-only and coupled-ocean configurations, there is no detailed documentation. Further study of model perturbation of statistics should be undertaken within a single-model framework.

### Change in seasonal variation under the warming

There is evident seasonal variation in lightning activity^46,47^, and the possible effects of future warming on such variation are of interest but poorly documented. In this context, particular attention should be paid to polar regions, as the distinct occurrence of day and night cycles throughout the year in such regions provides an excellent opportunity for study of seasonal changes^48^.

**Figure 4:**
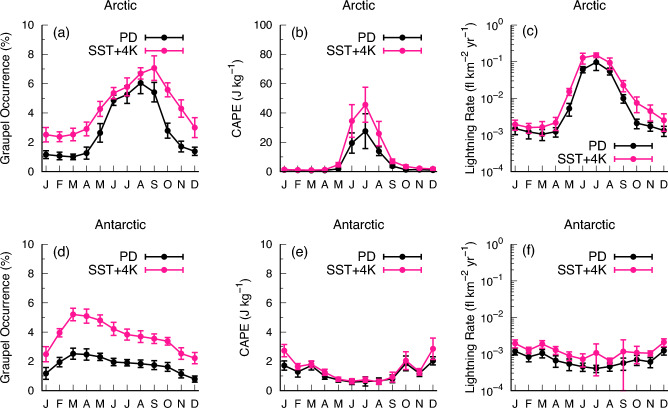
Monthly mean annual cycles of (**a**,**d**) graupel occurrence (%); (**b**,**e**) CAPE ($\textrm{J}\,\textrm{kg}^{-1}$); and (**c**,**f**) lightning flash rate ($\textrm{fl}.\,\textrm{km}^{-2}\,\textrm{yr}^{-1}$) over the Arctic (**a**–**c**) and Antarctic (**d**–**f**). The PD simulation is in black and the SST + 4K simulation in red. The scale in (**e**) is one-tenth that in (**b**). The error bars represent ±1 standard deviation of interannual variability.

Monthly time series of simulated graupel, CAPE, and lightning rate over the Arctic and Antarctic are shown in Fig. 4. In the Arctic region, graupel occurrence is higher in summer, particularly during June–September, and is lower in winter (Fig. 4a). This seasonality seems to be synchronized with that of CAPE (Fig. 4b) and therefore the resultant lightning flash rate (Fig. 4c), which is consistent with observational studies^47,49^.

Of note, although differences in CAPE between the PD and SST + 4K experiments were found only in their absolute values (Fig. 4b), differences in graupel occurrence between these experiments were also found in their seasonality (Fig. 4a) by shifting the maximal value to later in summer. Considering the relatively large difference in lightning flash rate between experiments for the period September–November (Fig. 4c), even though the CAPE did not change significantly, lightning activity must be influenced mainly by changes in graupel occurrence.

Such seasonal variation in graupel is also evident in the Antarctic (Fig. 4d) during February–April, but lightning rarely occurs poleward of 65^∘^ S^5,50^ due to the very stable environmental conditions in this region (Fig. 4e,f). Nevertheless, the Antarctic may be exposed to increased lightning under global warming (+ 26.4$\%\,\textrm{K}^{-1}$).

The results indicate that a reliable representation of seasonality in lightning activity under a global warming scenario is difficult to achieve unless the model considers the link between graupel and lightning, thereby emphasizing the need for the inclusion of prognostic graupel in GCMs. Furthermore, lightning occurrence may also be controlled by aerosol loading and type^38,49^. An understanding of interactions between microphysical aspects and lightning morphology at a fundamental process level^39,51^ will be crucial in future studies.

## Discussion

The response of lightning flash rate to aerosol perturbations and future global warming was considered using the MIROC6-CHIMERRA GCM. Most GCMs struggle to simulate lightning activity because graupel is ignored-a longstanding hindrance in lightning evaluation. Microphysical parameterization in CHIMERRA was therefore updated here by the inclusion of prognostic graupel.

The new microphysics scheme provided graupel distributions more consistent with those of cloud-resolving models and GPM/DPR satellite retrievals, and lightning flash rates consistent with LIS/OTD satellite observations (Figs. 1, 2). The global mean lightning flash rate is insensitive to PI through PD aerosol perturbations (+ 7.1%), but the impact of future warming on lightning activity is more evident. The global mean lightning rate is predicted to increase by 18.4$\%\,\textrm{K}^{-1}$, more than in earlier studies^13,16,42^.

The increased lightning rate in the present model is attributed to destabilization and more frequent graupel, with the latter predominating at higher latitudes (Fig. 3 and Supplementary Fig. 3; Table 1). In particular, the lightning rate in the Arctic is strongly dependent on the seasonality of graupel (Fig. 4). The month of graupel maximum occurrence is delayed to late summer in response to future warming, resulting in a larger difference between PD and future climate during the autumn season (Fig. 4c). Changes in lightning rate in different climates associated with this seasonality would not be captured by a simplified lightning scheme depending only on cloud-top height, thereby emphasizing the need for improved ice microphysics in GCMs. Although some GCMs have included prognostic precipitation for large-scale condensation^21,22,52^, cumulus precipitation is still treated as diagnostically, and graupel and hail are generally not represented in GCMs. Future work on improving lightning projections will also lead to an improved understanding of lightning-produced $\textrm{NO}_{\textrm{x}}$^53^ and lightning-ignited wildfires^9^.

Variations among models are likely attributable to model performance in simulating cloud vertical structures, different treatments of convective clouds, and different microphysics process representations^16–18,54^. Therefore, model and scheme intercomparisons should be undertaken to elucidate the sources of uncertainty at the fundamental process level, with fixed model configurations. The present study may provide a benchmark for further experiments with different model configurations (e.g., involving $\textrm{CO}_{\textrm{2}}$ and aerosol emission scenarios, different horizontal/vertical resolutions, and atmosphere-only or ocean-coupled scenarios), microphysics, and lightning schemes. Overall, the degree of confidence in simulating future responses of lightning to global warming depends on prognostic graupel parameterization being included in microphysics–lightning interactions.

## Methods

### MIROC6 with CHIMERRA prognostic graupel parameterization

The latest version of the MIROC6 global aerosol–climate model was used. This differs from the same version in the Coupled Model Intercomparison Project Phase 6 (CMIP6)^55^ in that it incorporates prognostic precipitation^22^. The model includes a two-moment large-scale microphysics scheme, the Cloud-Hydrometeors Interactive Module with Explicit Rain and Radiation (CHIMERRA), which considers both the mass and number mixing ratios of rain and snow. Precipitating hydrometeors also impact the radiation budget, so precipitation was radiatively active in the present model^34^. However, high-density ice hydrometeors (graupel and hail) are still not prognostic variables, which has been an obstacle to investigations of local orographic precipitation^29^ and the link between graupel and lightning in GCMs.

Therefore, another version of the model was devised with prognostic graupel parameterization. The bulk graupel class was treated as a two-moment scheme, based on the following prognostic equations:

1 $$\begin{aligned} \frac{\partial q_{g}}{\partial t}= & {} - \frac{1}{\rho _{a}} \nabla \cdot (\rho _{a} \mathbf{{u}} q_{g}) - \frac{1}{\rho _{a}} \frac{\partial (\rho _{a} q_{g} v_{q_{g}})}{\partial z} + S_{q_{g}} , \end{aligned}$$

2 $$\begin{aligned} \frac{\partial N_{g}}{\partial t}= & {} - \frac{1}{\rho _{a}} \nabla \cdot (\rho _{a} \mathbf{{u}} N_{g}) - \frac{1}{\rho _{a}} \frac{\partial (\rho _{a} N_{g} v_{N_{g}})}{\partial z} + S_{N_{g}} , \end{aligned}$$

where $q_{g}$ and $N_{g}$ are the mass and number mixing ratios of graupel, respectively; $\rho _{a}$ is air density ($\textrm{kg}\,\textrm{m}^{-3}$); $\textbf{u}$ is the wind vector representing horizontal advection ($\textrm{m}\,\textrm{s}^{-1}$); $v_{q_{g}}$ and $v_{N_{g}}$ are mass- and number-weighted fall velocities ($\textrm{m}\,\textrm{s}^{-1}$), respectively, representing vertical sedimentation; and $S_{q_{g}}$ and $S_{N_{g}}$ are respectively the source and sink tendencies ($\textrm{s}^{-1}$) of graupel with respect to deposition and sublimation, collection among other hydrometeors (i.e., accretion and riming), and melting, based on Reisner et al.^56^. Graupel was radiatively inactive.

The fall speed of larger graupel/hail may be $>10$ $\textrm{m}\,\textrm{s}^{-1}$, which is computationally expensive during simulations of vertical sedimentation; the Eulerian solver requires short time-steps consistent with Courant–Friedrichs–Lewy (CFL) criteria. Therefore, the vertical sedimentation scheme was updated to a time-implicit precipitation sedimentation scheme^52^. The model was solved for microphysics with 60 s iterations^22^, allowing the sedimentation process to be solved at the same time-step, reducing computational costs.

### Lightning parameterization and satellite data

The new parameterization with prognostic graupel enabled an evaluation of the frequency of lightning activity on a global scale. Among the various types of lightning scheme, it was convenient to apply one that explicitly considers graupel variation in considering the link between lightning rate and graupel morphology.

A scheme was chosen that combines frozen ice hydrometeors in the air column, $\textrm{Q}_{\textrm{frz}}$ ($\textrm{kg}\,\textrm{m}^{-2}$), and CAPE ($\textrm{J}\,\textrm{kg}^{-1}$) to parameterize the lightning flash rate, flight ($\textrm{fl}.\,\textrm{m}^{-2}\,\textrm{s}^{-1}$), following He et al.^23^:

3 $$\begin{aligned} f_\mathrm {{light}} = \alpha Q_\mathrm {{frz}} \textrm{CAPE}^{1.3} \end{aligned}$$

where $\alpha$ is a constant, and set to $2.67 \times 10^{-16}$ and $1.68 \times 10^{-17}$ for land and ocean, respectively, as applied by He et al.^23^ for best performance among the various schemes; and $\textrm{Q}_{\textrm{frz}}$ is the column integrated precipitating ice hydrometeors (cloud ice, snow, and graupel) between the 0 ^∘^C ($z_{0}$) and −25 ^∘^C ($z_{-25}$) isotherm levels:

4 $$\begin{aligned} Q_\mathrm {{frz}} = \int \limits ^{z-25}_{z_{0}} (q_{i} + q_{s} + q_{g}) \rho _{a} dz \end{aligned}$$

where $q_{i}$, $q_{s}$, and $q_{g}$ are the mass mixing ratios ($\textrm{kg}\,\textrm{kg}^{-1}$) for cloud ice, snow, and graupel, respectively, which were prognosed in the model; and $\textrm{Q}_{\textrm{frz}}$ is a proxy for the charging rate due to collisions of graupel with other hydrometeors within clouds^57^. The parameterized lightning flash rate is highly variable, depending on CAPE and $\textrm{Q}_{\textrm{frz}}$ (Supplementary Fig. 5). Although $\textrm{Q}_{\textrm{frz}}$ includes cloud ice and snow in addition to graupel, the simulated lightning rate shows a good correlation with GWP (Supplementary Fig. 6).

For model validation, the Lightning Imaging Sensor and the Optical Transient Detector (LIS/OTD) are widely used^58,59^. This satellite dataset provides flash number density ($\textrm{fl}.\,\textrm{km}^{-2}$) with a $2.5^\circ \times 2.5^\circ$ spatial resolution, and was used here in developing a climatology for the available period of July 1995–February 2014.

### Experimental setup and evaluations

The following three sets of experiments were undertaken to investigate the sensitivity of lightning activity to aerosol perturbations and future warming: (1) a present-day (PD, year 2000) control experiment; (2) a pre-industrial (PI, year 1850) aerosol experiment; and (3) a future-warming experiment with a uniform 4 K increase in sea surface temperature (SST + 4K). Model resolution was $1.4^\circ \times 1.4^\circ$ with 40 vertical levels, and an atmosphere-only configuration with year 2000 boundary conditions and prescribed climatological SST and sea ice. The model time-step was 12 min. A suite of simulations was undertaken for 11 years, and the subsequent 10 years used for analysis.

The impact of graupel treatment on the aerosol–cloud interaction (ACI) was assessed through $\textrm{ERF}_{\textrm{aci}}$^60^ quantified in the PD and PI experiments as follows:

5 $$\begin{aligned} \textrm{ERF}_{\textrm{aci}} = \textrm{CRE}_{\textrm{PD}} - \textrm{CRE}_{\textrm{PI}} \end{aligned}$$

where CRE is cloud radiative effect under clean-sky conditions^61^ for the removal of contamination of aerosols (i.e., aerosol–radiation interactions or direct effects).

Future warming effects on graupel and lightning were evaluated by differences between experiments (1) and (3). Cloud, precipitation, and meteorological representations are highly model-dependent, so here the percentage change is reported in response to a unit surface air temperature perturbation ($\%\,\textrm{K}^{-1}$), as reported in previous studies^13,42^.

### Supplementary Information

Supplementary Information.

## Data Availability

LIS/OTD satellite data are available at https://cmr.earthdata.nasa.gov/search/concepts/C1995865015-GHRC_DAAC.html. ERA5 products can be obtained from https://doi.org/10.24381/cds.f17050d7. The GPM 2ADPR product is downloaded from JAXA Global Portal System (https://gportal.jaxa.jp/gpr/). Simulation data is made publicly available through Zenodo (https://doi.org/10.5281/zenodo.7988296).
