# Supplementary material for: Significant increase in graupel and lightning occurrence in a warmer climate simulated by prognostic graupel parameterization
Source: Sci Rep. 2024 Feb 16;14:3862. doi: 10.1038/s41598-024-54544-5 (PMC10873401; doi:10.1038/s41598-024-54544-5)
Supplement: Supplementary file 1 — Supplementary Information. [file 41598_2024_54544_MOESM1_ESM.pdf]

Supplementary Information of *Significant increase in graupel and lightning occurrence in a warmer climate simulated by prognostic graupel parameterization*

Takuro Michibata[1,*]

[1]Department of Earth Science, Okayama University, Okayama, Japan
[*]tmichibata@okayama-u.ac.jp

Contents of this file

- Supplementary Figure 1
- Supplementary Figure 2
- Supplementary Figure 3
- Supplementary Figure 4
- Supplementary Figure 5
- Supplementary Figure 6
- Supplementary Table 1

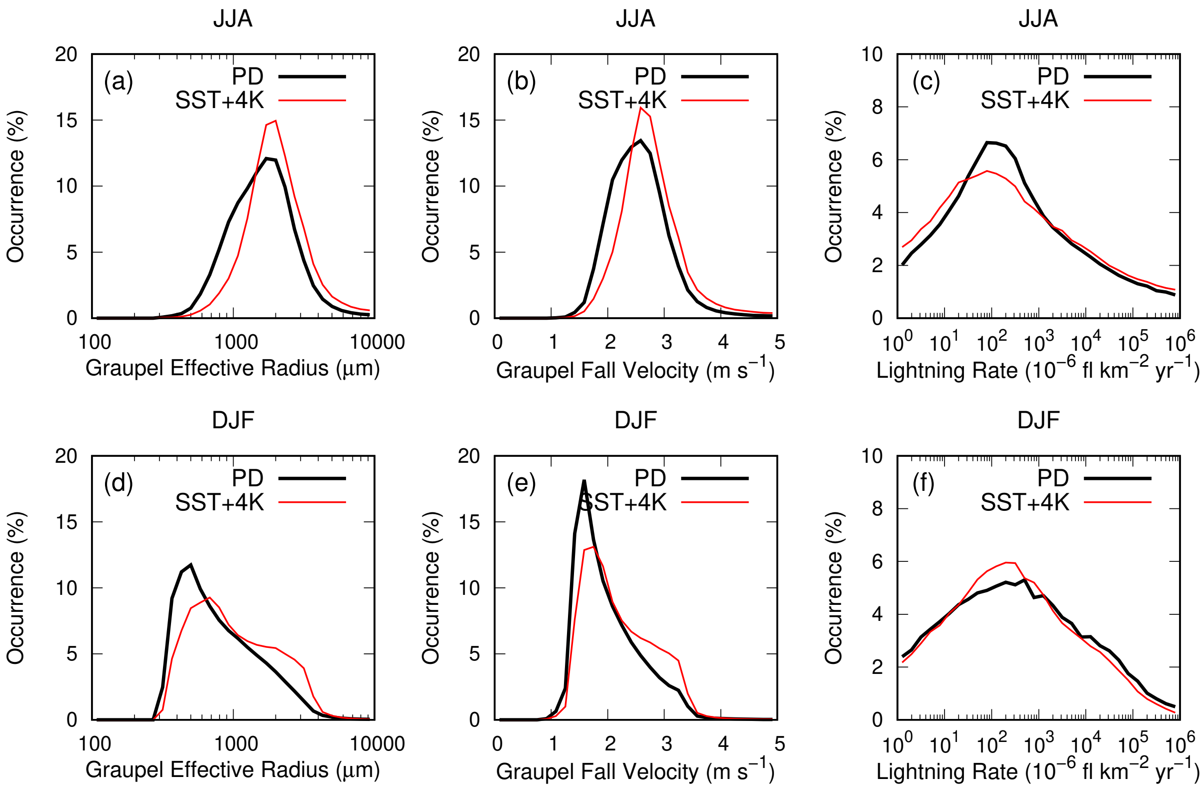

Supplementary Figure 1. Occurrence frequency of (a, d) graupel effective radius at the surface (µm); (b, e) graupel fall velocity at the surface (m s^{-1}); and (c, f) lightning flash rate (10^{-6} fl. km^{-2} yr^{-1}) for June–August (top) and December–February (bottom), above 66°N.

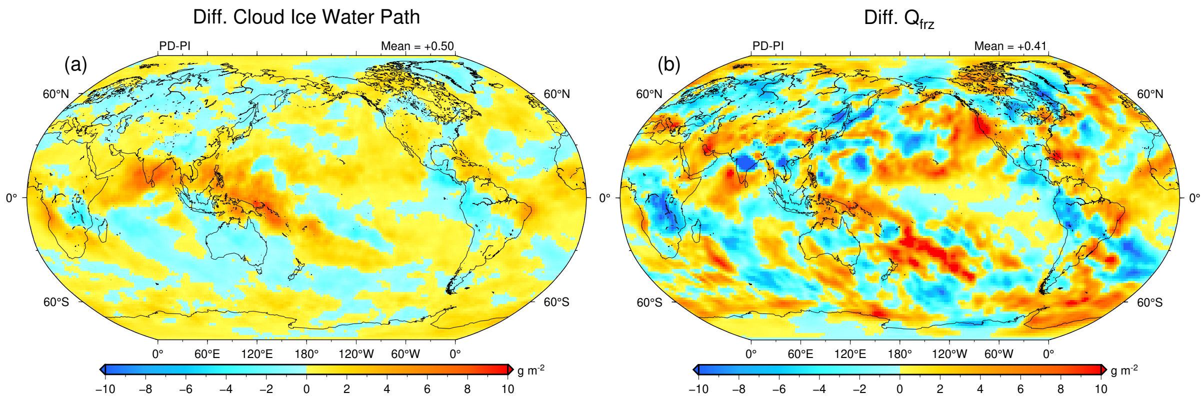

Supplementary Figure 2. Simulated changes in (a) the cloud ice water path (CIWP; $\mathrm{g\,m^{-2}}$) and (b) the precipitating ice water path (Q_{frz}; $\mathrm{g\,m^{-2}}$) from the pre-industrial era (PI, year 1850) to the present-day (PD, year 2000).

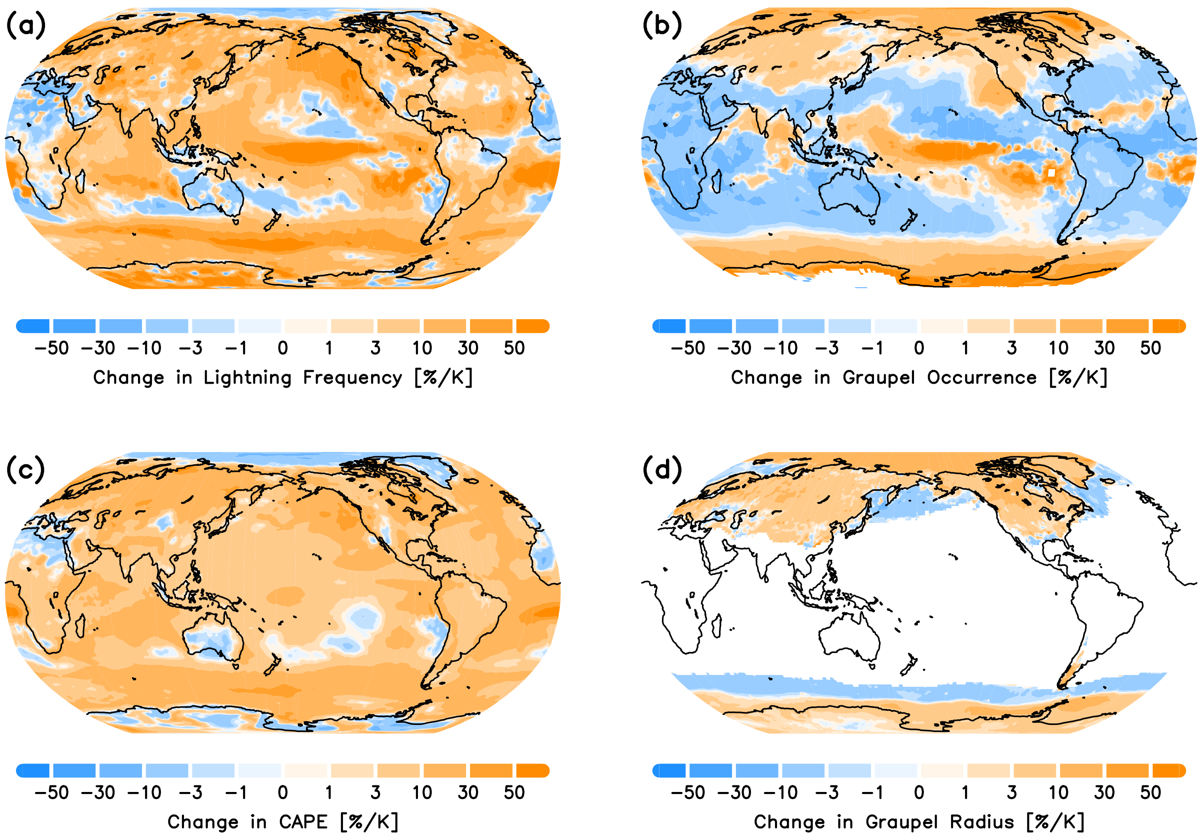

Supplementary Figure 3. Global distributions of future changes relative to PD ($\%\,\mathrm{K^{-1}}$) in (a) lightning flash rate, (b) graupel occurrence, (c) CAPE, and (d) graupel effective radius.

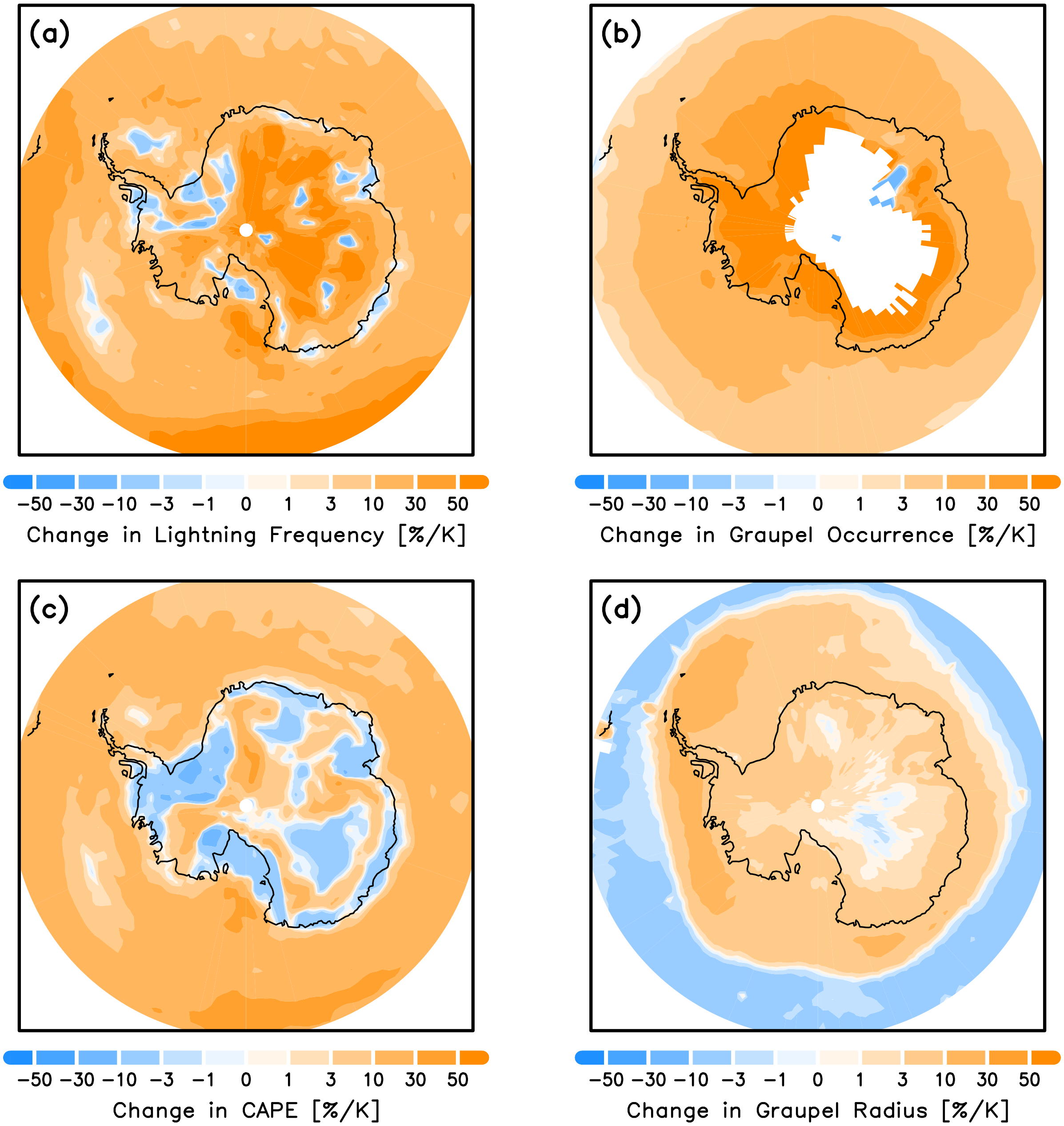

Supplementary Figure 4. Spatial distributions (below 55°S) of future changes relative to PD ($\%\,K^{-1}$) in (a) lightning flash rate, (b) graupel occurrence, (c) CAPE, and (d) graupel effective radius.

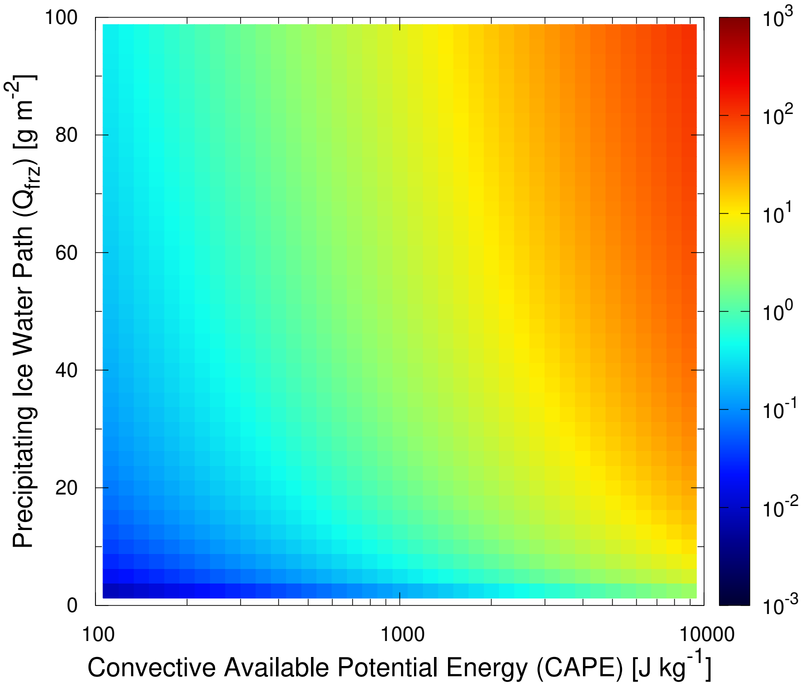

Supplementary Figure 5. Lightning flash rate (color scale; fl. $km^{-2}\,yr^{-1}$) as a function of convective available potential energy (CAPE; $J\,kg^{-1}$) and the precipitating ice water path (Q_{frz}; $g\,m^{-2}$), assuming a land area based on Equation (3).

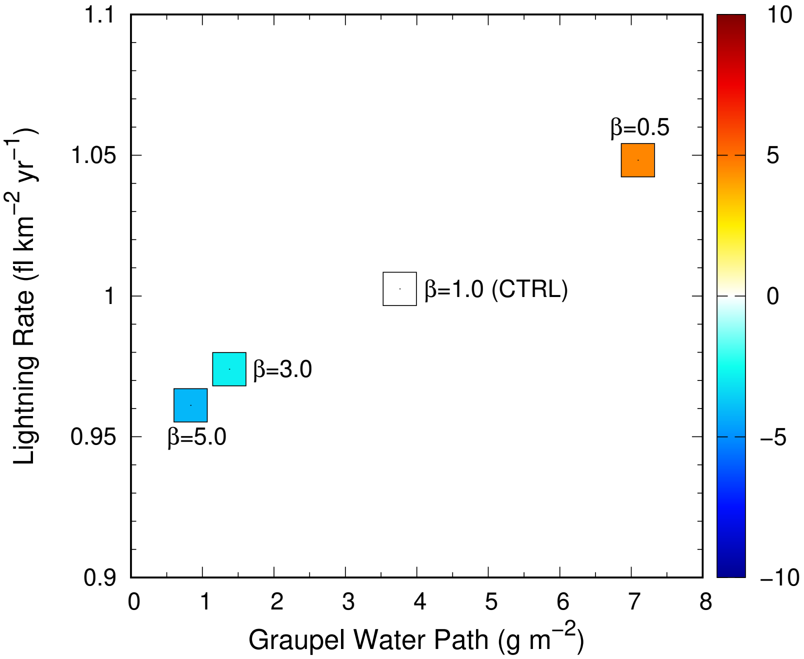

Supplementary Figure 6. Global mean lightning flash rate as a function of the global mean graupel water path (GWP; $g\,m^{-2}$) for the present-day (PD; year 2000) control simulation and three sensitivity experiments in which the fall velocity factor for graupel (β) is perturbed. The color scale indicates the percentage difference of the global mean lightning rate relative to the control simulation (CTRL; β = 1.0).

Supplementary Table 1. Global mean values for the key parameters used in this study. Note that the percentage change listed in Table 1 is not obtained from the values in this table because the data in Table 1 are obtained from the mean percentage change at each grid box, normalized to the increase in global mean surface temperature.

	PI (year 1850)	PD (year 2000)	Future (SST+4K)
Lightning Rate (fl. $km^{-2}\,yr^{-1}$)	1.0	1.0	1.3
CLWP[a] ($g\,m^{-2}$)	83.7	83.9	94.8
CIWP[b] ($g\,m^{-2}$)	16.1	16.6	21.0
SWP[c] ($g\,m^{-2}$)	55.1	55.3	55.7
GWP[d] ($g\,m^{-2}$)	3.7	3.8	3.9
Q_{frz}[e] ($g\,m^{-2}$)	49.6	50.0	52.8
Graupel Occurrence (%)	4.6	4.6	4.5
Graupel Radius (mm)	1.7	1.7	2.0
CAPE[f] ($J\,kg^{-1}$)	462	461	625

[a]CLWP: Cloud Liquid Water Path.
[b]CIWP: Cloud Ice Water Path.
[c]SWP: Snow Water Path.
[d]GWP: Graupel Water Path.
[e]Q_{frz}: Total precipitating ice hydrometer [see Eq.(4)].
[f]CAPE: Convective Available Potential Energy.
